# End-of-life patients and palliative care in intensive care units in Croatia: the current situation

**DOI:** 10.3325/cmj.2023.64.140

**Published:** 2023-04

**Authors:** Diana Špoljar, Dinko Tonkovic

**Affiliations:** 1 University of Zagreb School of Medicine, Zagreb, Croatia; dianaspoljar@gmail.com; 2 Zagreb University Hospital Center, Zagreb, Croatia

Decisions about the limitation of life-sustaining treatments (LST) in end-of-life patients are becoming more frequent in intensive care units (ICUs) (1). In close connection to these decisions is the provision of adequate palliative care.

In 2021, the Croatian Ministry of Health issued the Guidelines for Quality Improvement of Palliative Care in ICUs (2). These guidelines state that in end-of-life patients all futile treatment should be limited and that personalized palliative care should be provided. Analgesic and sedative therapy should be administered in doses that alleviate pain and suffering, despite the fact that it might hasten the patient’s death. Attention should be paid to the wishes of the patients and their family members, who are to be given psychological support. The patient and the family need to be involved in the decision-making process and informed about all treatment options and possible outcomes in a way they find understandable.

The Croatian Health Care Act defines the provision of palliative care as one of the fundamental aspects of health care. However, it also states that patients must receive all diagnostic and therapeutic procedures when their health or lives are endangered, without any caveats for end-of-life patients (3). This puts ICU medical professionals in an unenviable position as they balance between patients’ wishes, best medical practices, legal obligations, and their own moral principles.

Before the publication of the guidelines, Croatian scientists conducted an extensive research project investigating the values and decisions in end-of-life situations. A part of the project was a study exploring the attitudes and experiences of physicians and nurses treating end-of-life patients in ICUs (4). According to the study, decisions on LST limitation were not frequently made. Inotropes and antibiotics were more commonly withdrawn than was mechanical ventilation. Decisions were made by physicians and rarely by nurses. Family members were included in the decision-making process in about half of the cases. In general, the results showed a prevalence of paternalistic and conservative attitudes, which were also observed in other countries in southern Europe (5).

In 2019, we conducted a prospective study to examine the decision-making process in the ICUs in real time. Questionnaires were administered to the directors of 15 ICUs in six tertiary hospitals in Croatia. Disappointingly, only five questionnaires from three hospitals were returned. The results showed that the discussion about the limitation of LST was initiated by physicians, who were also the ones making these decisions. Family members and nurses were involved in only two cases. All patients received palliative sedative and/or analgesic therapy and had some types of treatment withheld or withdrawn. Spiritual or psychological support were not provided to any of the patients. A palliative care specialist was involved in only one case (Table 1).

**Table 1:** Characteristics of patients and the decision-making process in a study enrolling end-of-life patients in the intensive care units (ICU) of tertiary hospitals in Croatia

	Patient 1	Patient 2	Patient 3	Patient 4	Patient 5
**Type of ICU**	Surgical	Surgical	Surgical	Surgical	Neurologic
**Patient’s age**	74	79	87	72	35
**Main diagnosis**	Sepsis	Sepsis	Multi-organ failure	Respiratory insufficiency	Bacterial meningitis
**Comorbidities**	Yes	Yes	Yes	Yes	No
**Cardiopulmonary resuscitation performed**	No	No	No	No	Yes
**Initiator of decision-making**	Physician	Physician	Physician	Physician	Physician
**Involved in decision-making**	Physician, nurse	Physicians (anesthesiologist and surgeon), nurse	Physicians (anesthesiologist and surgeon)	Physician	Physician
**Family involved**	Yes	No (no known family)	No	No	Yes
**Days passed between admission and decision to withhold/withdraw treatment**	30	17	2	10	2
**Withheld treatment**	Antibiotics, inotropes, vasoactive therapy	Inotropes, vasoactive therapy	Nutrition, surgery	na	Antibiotics, vasoactive therapy, inotropes, mechanical ventilation
**Withdrawn treatment**	Hemodialysis	n/a	Inotropes, vasoactive therapy, antibiotics	Nutrition, inotropes, vasoactive therapy	Nutrition, hydration, blood products transfusion
**Palliative sedative therapy**	No	No	Yes	Yes	Yes
**Palliative analgesic therapy**	Yes	Yes	Yes	Yes	No
**Involvement of palliative care specialist**	No	Yes	No	No	No
**Spiritual or psychological support**	No	No	No	No	No

A very low response rate obtained in this study could be explained by the rigidity of the current legal framework in Croatia. As the law does not explicitly permit the limitation of LST in end-of-life patients, certain physicians may have been reluctant to participate in studies evaluating such actions. Also, this research indicates that the treatment of end-of-life patients in Croatia is not a very prominent issue. The issue has also not been sufficiently addressed by the official associations or the public, which leads to a paternalistic approach. There is a need for further research of this type with a higher participation rate, so that more representative data can be obtained.

As shown in the mentioned studies, cases of LST limitation were occurring in Croatian ICUs before proper guidelines or an adequate legal framework were in place. Physicians working with end-of-life patients, recognizing the specifics of this type of treatment, acted according to the current medical expertise and recommendations of Western countries, which emphasize the patient's autonomy and dignity (6).

The guidelines of the Croatian Ministry of Health state that LST limitation decisions should be made within the medical team caring for the patient, but they do not openly state that nurses should be included (2). This may pose a problem, as studies indicate a lack of nurse involvement. Since nurses are an indispensable part of the medical team, the guidelines should clearly affirm their inclusion in the decision-making process, even though the final decision is made by the treating physician.

Medical professionals providing palliative care and treating end-of-life patients face many difficulties. Some of these difficulties pertain to moral principles and some to technical and logistic aspects of care. This complex issue cannot be resolved easily, as it involves different types of people, professions, conditions, cultural values, and backgrounds. However, a structured and organized approach to the treatment of end-of-life patients and the provision of palliative care leads to a higher satisfaction for the patients and their family members, and diminishes the moral distress of medical professionals (7). The first step in the right direction was the publication of guidelines providing instructions and systematic support for medical professionals dealing with challenging situations. A further step should be the establishment of the Croatian legal framework. If we are to provide high-quality care to palliative and end-of-life patients, the law and regulations must create a safe environment for the medical professionals and the patients. Furthermore, medical professionals working with end-of-life patients should receive proper education. Encouraged by the publication of guidelines, we strongly believe that further positive changes benefiting patients and medical professionals will emerge from sustained effort and continuous work.
